# Takayasu Arteritis Presenting as Intestinal Angina: Unusual Presentation of a Rare Disease

**DOI:** 10.7759/cureus.21548

**Published:** 2022-01-24

**Authors:** Pankaj Nawghare, Ravi Thanage, Shubham Jain, Sanjay Chandnani, Pravin M Rathi

**Affiliations:** 1 Gastroenterology, BYL Nair Hospital, Mumbai, IND

**Keywords:** anticoagulant, absent pulse, aneurysm, claudication, abdominal pain

## Abstract

Intestinal angina refers to abdominal pain resulting from reduced mesenteric blood flow. Although atherosclerosis is the most common cause, large vessel vasculitis is emerging as one of the common causes. We have reported a case of an 18-year-old female who presented with classical symptoms of intestinal angina. On evaluation, the patient was found to have an abdominal aortic aneurysm with a compromised mesenteric blood supply. She was started on steroids and methotrexate along with anti-platelets and anticoagulants. She improved following the treatment and didn't have any complaints on follow-up.

## Introduction

Takayasu arteritis (TAK) is an inflammatory disease of large and medium sized arteries that usually affects middle-age female of Asian descent. There is considerable variation in disease expression. However, the initial vascular lesions frequently occur in the aorta and its main thoracic branches. Although mesenteric artery involvement is common in TAK, initial presentation as intestinal angina is rare. We present a case of chronic mesenteric ischemia in a patient of TAK presented with post-prandial pain, which is an uncommon presentation of TAK and its exact occurrence is not well defined in current literature.

## Case presentation

An 18-year-old female presented with complaints of abdominal pain for the last three months, which was upper abdominal, dull aching in nature, intermittent starting within the first hour of meal lasting up to 2-3 hours post-meal and relieves on its own. There was no history of vomiting, altered bowel habits, or gastrointestinal blood loss. She also had lost weight approximately 10 kgs in the last three months due to fear of abdominal pain after eating and claudication in both lower limbs on exertion. In the past, she had a history of right lower limb deep venous thrombosis three years back, for which she took anticoagulants for six months. She also had a history of convulsion six months back, diagnosed to have cortical venous sinus thrombosis (CVST), and she was started on anticoagulant and anticonvulsant medicines. She denied a history of any addiction. She also denied a history of any other drug intake, including oral contraceptive pills. There was no history of similar complaints in the family.

On general examination, she was found pale. Her femoral, popliteal, and dorsalis pedis pulsation were absent on both sides. The rest of the peripheral pulses were normal. Blood pressure in the upper limb was 110/70 mmHg, while in the lower limb it was non-recordable. Per abdominal examination revealed a pulsatile epigastric lump of size 3.5 cm X 5 cm and on auscultation bruit was heard. The rest of the systemic examination was unremarkable.

Her blood investigations on admission showed hemoglobin 6.9 g/dl, leucocyte count 13,800/µl, platelets 6,98,000/µl, and C-reactive protein 45 mg/l. The rest of the routine blood investigations were normal. The serum iron study was suggestive of iron deficiency anemia. Stool routine microscopy was suggestive of positive occult blood with 4-5 RBC's/high power field (HPF). Abdominal radiogram and esophagogastroduodenoscopy were unremarkable. Lower limb Doppler was suggestive of reduced flow in bilateral femoral, popliteal, and dorsalis pedis artery with no evidence of any thrombus or stenosis.

**Figure 1:**
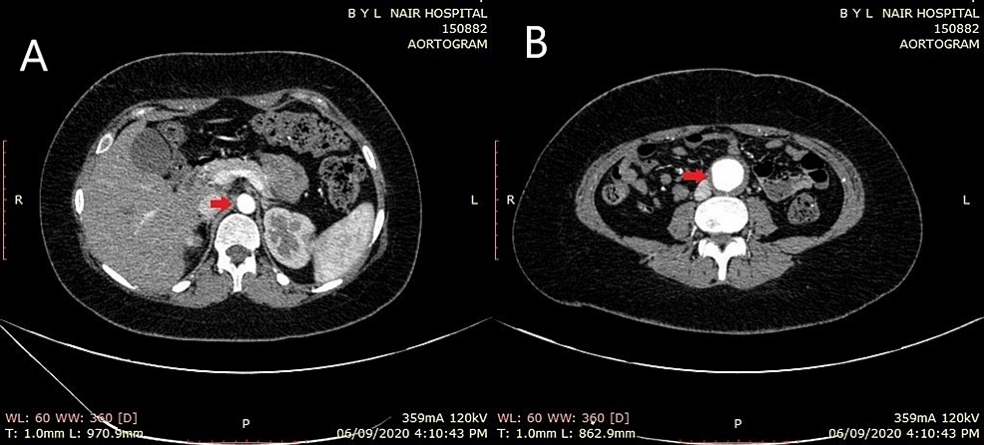
CT aortogram (transverse section) CT images showing dilated infrarenal aorta (red arrow) measuring 3 cm X 3.2 cm (B) as compared to the normal suprarenal aorta (red arrow) measuring 1.2 cm X 1.4 cm (A)

**Figure 2:**
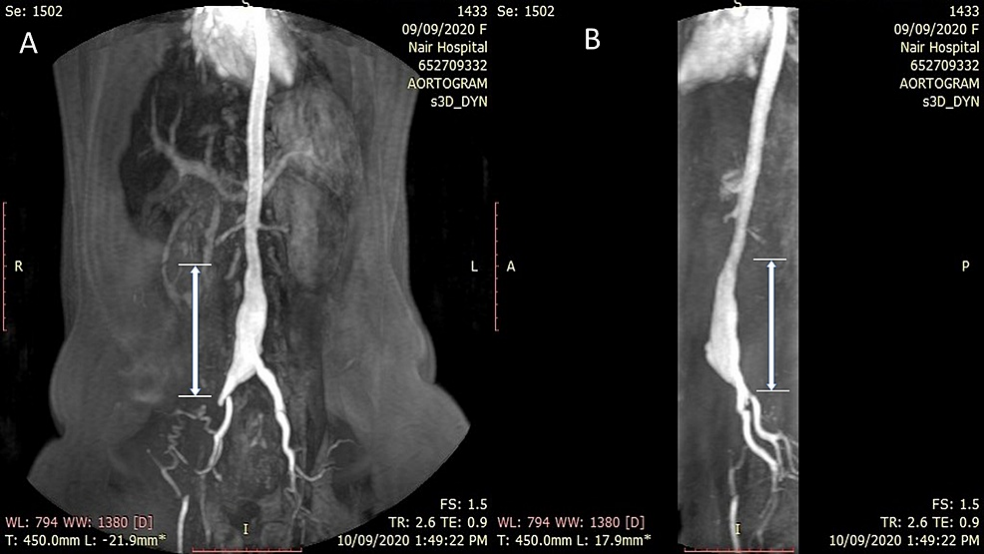
CT aortogram Coronal (A) and sagittal (B) section showing long segment (vertical extent-7.9 cm) fusiform dilatation of infrarenal part of the abdominal aorta

Ultrasonography of the abdomen revealed dilatation of the infrarenal abdominal aorta measuring 3 cm in diameter with hypoechoic areas within. Subsequently, computerized tomography (CT) aortogram was done, which showed dilated infrarenal aorta of size 3 cm X 3.2 cm, for a length of 7.9 cm till its bifurcation into the bilateral common iliac arteries (Figures 1-2). Her CT scan also revealed non-enhancing complete lumen occluding thrombi in bilateral external iliac arteries, superior and inferior mesenteric arteries. Other large vessels were normal. However, there was no evidence of bowel ischemia on a CT scan of the abdomen.

**Figure 3:**
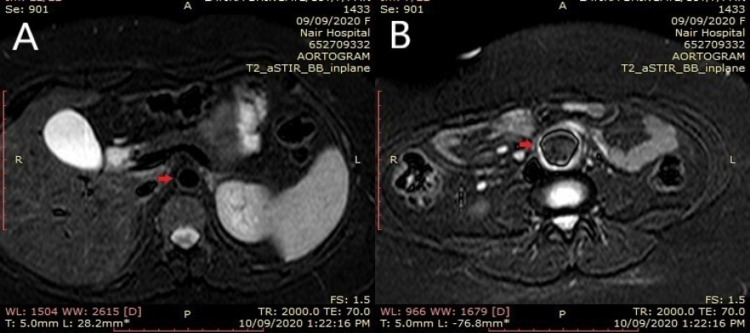
MRI aortogram MRI images showing wall thickness (red arrow) and enhancement in the suprarenal part (B) as compared to the normal aorta (red arrow) (A)

Rheumatologist opinion was taken for concern about large vessel vasculitis. Keeping the possibility of Takayasu arteritis, MRI aortography was advised to see the activity of the disease. MRI aortogram revealed post-contrast enhancement in the suprarenal aorta and proximal superior mesenteric artery (SMA) suggestive of active disease (Type IV Takayasu arteritis, Numano classification) (Figure 3). However infrarenal part shows multiple wall calcification suggestive of chronic aortoarteritis.

Thrombophilia workup was done because of multiple thrombi which came negative. She was managed conservatively. Blood transfusion was done to correct anemia and an iron supplement was started. She was given tablet prednisolone 1 mg/kg/day for four weeks followed by slow tapering over the next two months to 20 mg/day for maintenance. Tablet methotrexate 15 mg weekly was also added. Anticoagulant which was previously started for CVST was continued with a therapeutic international normalized ratio (INR) target range maintained between 2 to 3. Aspirin 75 mg once a day was also added. Anticonvulsant was continued. However, even after one month of therapy patient symptoms didn't improve. Hence, she was transferred to the surgical unit for surgical intervention in view of persistent ischemic symptoms.

## Discussion

Chronic mesenteric ischemia also referred to as intestinal angina, is a rare condition, characterized by postprandial abdominal pain. It occurs due to reduced blood flow of the small intestine that is usually seen in patients with multivessel mesenteric stenosis or occlusion. Atherosclerosis is the most common cause, other causes include fibromuscular dysplasia, polyarteritis nodosa, and TAK. TAK is a form of large vessel granulomatous vasculitis of unknown etiology. Young women in the second and third decades of life are usually affected [1]. The disease has a worldwide distribution, with the greatest prevalence among Asian females [2].

Although the pathogenesis of TAK is not fully known, the disease likely results from an inflammatory response in a genetically prone individual. The disease is characterized by inflammatory granulomatous vasculitis of medium and large arteries, which leads to transmural fibrosis and thickening of the arterial walls, leading to arterial stenosis, thrombosis, aneurysms, and eventual ischemic changes [3]. The onset of symptoms is sub-acute, causing a delay in diagnosis that can range from months to years, during which time the vascular disease may start and progress to become symptomatic. It is not uncommon for the consequences of the arterial disease to be the first sign of TAK noticed at presentation as in this case.

The clinical manifestation of TAK varies according to the time point along the disease course. The early active inflammatory stage is characterized by non-specific systemic symptoms like malaise, fever, night sweats, weight loss, joint pain, fatigue, and fainting. The disease course may follow a remitting/relapsing pattern during this stage, making the diagnosis difficult [4,5]. The recurrent disease usually appears in new arterial territories, with the consequent coexistence of active and inactive (sequelae) lesions. The late, chronic phase (the "pulseless" stage) is characterized by vascular insufficiency, ischemia, and symptoms secondary to arterial occlusion.

The aortic arch is more involved in Japan while the involvement of the abdominal aorta is more in Indian and Korean patients [6]. Visceral arterial involvement is common and is reported in 11 to 68% of patients, usually in the form of steno-occlusive lesions [7]. The disease usually involves the celiac artery and superior mesenteric artery [7]. The unevolved inferior mesenteric artery provides a collateral network in patients with SMA obstruction. So, the obstructive lesions in the mesenteric circulation are usually clinically silent due to abundant collateral supply. Simultaneous involvement of SMA and inferior mesenteric artery (IMA) is a rare occurrence in TAK, as in our patient. Such patients may present with intestinal angina or mesenteric vascular ischemia. So, chronic intermittent postprandial abdominal pain can be an initial presenting feature of mesenteric ischemia in TAK patients [8]. Severe TAK complicated by mesenteric infarction, on the other hand, is rarer. The simultaneous occurrence of the CVST and TAK is a rare finding in our case. Nogueira et al. had reported a case of TAK with CVST [9].

Management of TAK is often challenging. Treatment of TAK is aimed at controlling vascular inflammation and stopping all injuries due to vasculitis. Inactive disease without medication is rare in TAK patients and the disease may progress even during clinically inactive phases of the disease [10]. The European Alliance of Associations for Rheumatology (EULAR) 2018 recommendations for the management of TAK advises induction of early remission with corticosteroids and use of immunosuppressive agents as adjunctive therapy [11]. Commonly used immunosuppressive agents include azathioprine, methotrexate, mycophenolate mofetil, leflunomide, and cyclophosphamide [12]. Vascular surgery has a relevant adjunctive role, usually in medically refractory cases. However, elective endovascular interventions or surgery are performed during stable remission. But conditions like arterial vessel dissection or critical vascular ischemia require urgent intervention [11]. The major advantage of endovascular therapy is its minimally invasive nature and shorter hospital stay. Its disadvantages are the need for reintervention and inferior durability [13].

## Conclusions

A high index of suspicion for Takayasu arteritis must be kept while evaluating chronic mesenteric ischemia in young females presenting with postprandial abdomen pain and having asymmetrical pulses. Early diagnosis and timely intervention are critical for preventing morbidity and mortality.
